# NAC in the middle: the conserved NAC transcription factor OsCUC1 controls organ boundary formation in rice

**DOI:** 10.1093/plcell/koag157

**Published:** 2026-06-01

**Authors:** Pablo González-Suárez, Yanjie Song

**Affiliations:** Assistant Features Editor, The Plant Cell, American Society of Plant Biologists; Department of Developmental Genetics, Centre for Plant Molecular Biology (ZMBP), Eberhard Karls University, Tuebingen D-72076, Germany; School of Biology, University of Leeds, Leeds, West Yorkshire LS2 9JT, United Kingdom

Most seed plants spend a considerable part of their lifetime generating new organs, including leaves, shoots, and eventually flowers. These arise from meristems, which harbor pools of undifferentiated stem cells with the potency to form new cell types and tissues. Meristem/organ boundaries, which lie between a meristem and the newly formed organ, are key for successful organogenesis. Accordingly, their establishment is tightly regulated in the plant kingdom by conserved NAC transcription factors of the CUP-SHAPED COTYLEDON (CUC) family (Žádníková and Simon 2014). Within organs, boundary regions also play a critical role in separating distinct cell and tissue types, such as the collar, blade, and sheath in cereal leaves or the abscission zone (Fig. 1a). While the genetic control of boundary regions has been extensively studied in dicots, the mechanisms underlying their establishment in monocots remain less explored.

**Figure 1:**
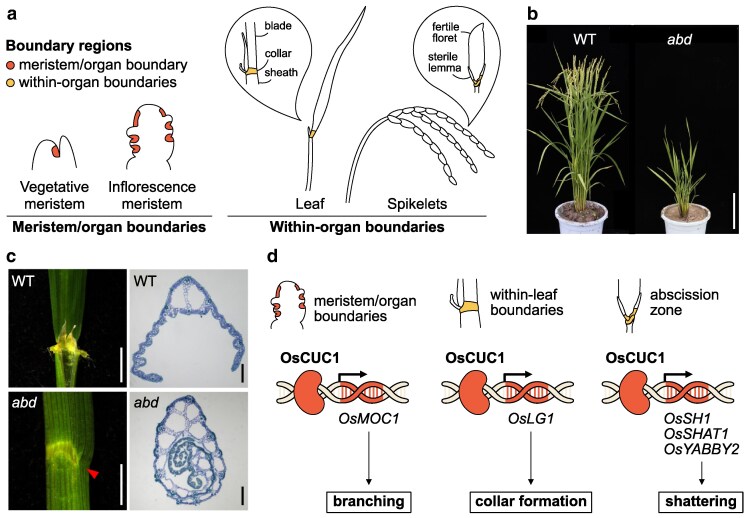
The NAC transcription factor OsCUC1 regulates organ boundary establishment in rice. a) Diagram highlighting different meristem/organ and within-organ boundaries in rice. b) Photographs from Li et al. (2026) illustrating the developmental phenotypes displayed by the *abd* natural rice mutant. c) Photographs and microscopy images depicting the defects in collar development in leaves of *abd* mutants. d) Schematics depicting the role of OsCUC1 in the establishment of distinct boundary regions revealed by Li et al. (2026). Figure credit: P. González-Suárez.

Recognizing the critical role of organ boundaries in cereal architecture, **Zeyu Li, Suikang Wang, Longjun Zeng, and colleagues** (Li et al. 2026) set out to dissect the genetic basis of boundary development in rice. Their work began with the identification of a natural rice mutant, *abnormal boundary development* (*abd*), which displayed profound defects in panicle branching and leaf collar formation (Fig. 1, b and c). Map-based cloning uncovered the gene underlying the *abd* phenotype, *OsCUC1*, an ortholog of the Arabidopsis NAC transcription factors CUC1/2, which had been previously implicated in rice boundary regions (Chang et al. 2021; Wang et al. 2021). Introducing *OsCUC1* in the *abd* mutant background, the authors were able to rescue the developmental phenotypes, confirming a causative role for *OsCUC1*.

Notably, phenotypic analysis of CRISPR/Cas9-generated *OsCUC1* mutants revealed partial functional redundancy with its homeolog, *OsCUC3*. *OsCUC1* knockout mutants exhibit strong developmental defects similar to the *abd* mutant, while *oscuc3* mutants showed some overlapping effects. Moreover, analysis of gene expression demonstrated that *OsCUC1* and *OsCUC3* are specifically expressed in meristem/organ boundary regions, pointing to a defect in their establishment as the likely cause of the mutant phenotypes. In leaves, *OsCUC1* is also expressed in the leaf blade/sheath boundary region, prompting the authors to investigate an additional role in within-organ boundary regions. Interestingly, *OsCUC1* overexpression lines showed enhanced collar formation, while *abd* mutants display collar domain loss, suggesting that *OsCUC1* is essential for the establishment of the leaf blade/sheath boundary.

Combining transcriptomic profiling and chromatin immunoprecipitation sequencing, the authors aimed to identify transcriptional targets of OsCUC1, which they extensively validated using molecular and biochemical assays. Among them was *MONOCULM1* (*MOC1*), a key regulator of axillary meristem initiation and tillering, which is directly activated by OsCUC1 to regulate branching. In agreement with this, disruption of the binding site of OsCUC1 in the *MOC1* promoter led to a reduction in branching. Using similar approaches, the authors identified additional targets of OsCUC1 in different developmental stages and tissue types, including LIGULELESS1 (*OsLG1*), which regulates collar formation in the leaf, and the shattering-related genes QTL FOR SEED SHATTERING ON CHROMOSOME 1 (*qSH1*), SHATTERING ABORTION1 (*SHAT1*), and *OsYABBY2*, which control the shattering of sterile lemmas.

Through the analysis of genes induced by *OsCUC1* overexpression, the authors identified several putative targets associated with signaling of hormones, such as auxin and brassinosteroids. In light of the characterized role of hormones in boundary formation in Arabidopsis (Gendron et al. 2012), this positions *OsCUC1* as a potential central regulator that integrates hormonal signaling with boundary development in rice.

The establishment of boundary regions is critical for both organ formation and within-organ tissue separation, thereby contributing greatly to plant architecture. Boundary regions therefore influence tiller number, leaf shape, and leaf angle, which are central factors that determine crop yield. Through their findings, Li et al. (2026) provide a deeper insight into how the NAC transcription factor *OsCUC1* controls plant architecture, revealing a complex regulatory network upstream of multiple regulators across rice development (Fig. 1D). Their work not only advances our understanding of boundary development in monocots but also provides potential targets that can be manipulated to optimize plant architecture and enhance the yield potential of cereal crops.

## Recent related articles in *The Plant Cell:*

- He et al. (2024) show that the polarity regulators OsKANADI1 and OsYABBY5 regulate gibberellin metabolism to control aerial architecture in rice.
- Overholt et al. (2026) demonstrate that CUC3 is under the regulation of EPIDERMAL PATTERNING FACTOR-LIKE2 (EPFL2) during ovule development in Arabidopsis.

## Data Availability

No new data were generated or analysed in support of this research.
